# Assessing different models to predict the growth and development of pepper plants under water deficits

**DOI:** 10.3389/fpls.2024.1436209

**Published:** 2024-12-11

**Authors:** Jun Zhu, Yuanda Zhang, Guangxian Yang, Shuxian Liu

**Affiliations:** ^1^ Shangqiu Meteorological Bureau, Shangqiu, China; ^2^ Henan Key Laboratory of Agrometeorological Support and Applied Technique, China Meteorological Administration, Zhengzhou, China; ^3^ Institute of Henan Meteorological Sciences, Zhengzhou, China; ^4^ State Key Laboratory of Severe Weather, Chinese Academy of Meteorological Sciences, Beijing, China; ^5^ National Meteorological Center, China Meteorological Administration, Beijing, China

**Keywords:** pepper, water capacity, physiological development time, spline interpolation, phenology simulation

## Abstract

To construct pepper development simulation models under drought, experiments of water capacities of 45–55%, 55–65%, 65–75% or 75–85% and exposure (2, 4, 6 or 8 d) (Exp. 1 & 2), of 50–60%, 60–70% or 70–80% and exposure (3, 5, and 7 d) (Exp. 3) were conducted with “Sanying” pepper. Physiological development time (PDT), product of thermal effectiveness and PAR (photosynthetically active radiation) (TEP) and growing degree days (GDD) were used to simulate growth under various treatments in Exp. 1. Plant development was influenced by the severity and drought duration. Mild water deficits (65–75% for 2–6 d or 55–65% for 2–4 d) accelerated development, while severe water deficits (65–75% for 8 d, 55–65% for 6–8 d or 45–55% for 2–8 d) delayed development. The PDT gave the highest coefficient of determination (R^2^, 0.89–0.94) and the lowest root mean squared error (RMSE, average of 1.03–1.50 d) and relative error (RE, average of 1.60–1.88%) for simulating three growth periods (Exp. 2). It was therefore used to construct growth models under water capacity of 45–85% over 2–8 d with spline, cubic, makima, linear, and nearest interpolation. Validation in Exp. 3 indicated that the spline model was optimal, having the highest R^2^ (0.96–0.97) and the lowest RMSE (average of 1.31–1.75 d) and RE (average of 1.18–2.06%). The results of the study can help producers to optimize water management and to develop drought strategies for production.

## 1 Introduction

Pepper (*Capsicum annuum* L.) is an important greenhouse crop in China, and is rich in magnesium, iron, calcium, and zinc (Dai et al., 2022; Padilla et al., 2023a). It is a shallow rooted crop with thin and weak roots, and susceptible to drought (Zhang et al., 2023a). Water deficits can significantly affect pepper growth and development (Kurucz et al., 2023; Padilla et al., 2023b). In recent decades, many studies have been carried out on the effects of water deficits on growth and development in peppers (Sanogo, 2006; Peng et al., 2010; Zhu et al., 2012; Lv et al., 2019; Castronuovo et al., 2023). Yang et al. (2016) suggested that mild water deficits may not affect the growth of above-ground parts, while sever deficits can significantly reduce the shoot length and photosynthesis (Adnew et al., 2023; Molla et al., 2023), and had an impact on fruit yield (Admassie et al., 2022). Pan (2007) concluded that the optimal field water capacity is 80–90% based on a series of experiments. However, some studies suggest that short and long-term stress can cause different effects on crops (Widuri et al., 2020; Xu et al., 2020a; Zhang et al., 2023b). We were interested in studying the effects of different water deficit levels over different durations from a few days to a week or more.

Current studies on models of crop growth have been developed for several decades (Ahmad et al., 2017; Perera et al., 2020; Barriball et al., 2022; Didevarasl et al., 2023). Diao et al. (2009) simulated the development and yield of pepper using a product of thermal effectiveness and PAR (TEP). Torrion et al. (2011) simulated soybean growth in fields of the North-Central United States according to a soybean model (SoySim). Ma and Tian (2016) modelled phenology and leaf area of watermelon based on physiological development time (PDT) and logistic function, respectively. Chen et al. (2019) proposed a sugarcane development simulation model (SDSM) to predict growth of newly planted and perennial sugarcane based on clock model. Niu et al. (2021) used PDT and growing degree day (GDD) to simulate growth of greenhouse cherry tomatoes. Cai et al. (2021) modelled the plant nutrition and physiology in Chinese cabbage using light and temperature data. Leguízamo-Medina et al. (2023) simulated the growth and flowering of carnation based on cumulative GDD. Xu et al. (2020a) compared three models to simulate growth and yield in strawberry and recommended PDT method. Therefore, PDT, TEP and GDD had been widely used to model growth in crops (Chen et al., 2021; Shi and Li, 2021; Chen et al., 2022; Liu et al., 2022; Sun et al., 2022; Wang et al., 2023).

Current studies on the modeling of crop growth under water deficits have focused on a single orthogonal treatment. Interpolation can be used to predict values of growth between data of known points. These methods, including nearest, linear, cubic, spline, and makima, have different applications under various environmental conditions (Gore et al., 2023). For example, Chen et al. (2011) suggested that the surface interpolated by a spline method was smoother. Xiao et al. (2021) concluded that a cubic model is optimal to simulate temperature in a greenhouse.

This study compared PDT, TEP or GDD to model growth of pepper under water capacities of 45–55%, 55–65%, 65–75%, and 75–85% over 2, 4, 6 or 8 days. Then interpolation was used to construct a three-dimension models under water capacity of 45–85% over 2–8 days. Finally, another independent experiment of water capacity (50–60%, 60–70% or 70–80%) and treatment days (3, 5 or 7 d) was conducted to screen out the optimal interpolation model. We expected that the study could provide a scientific method for application of crop simulation models in agriculture.

## 2 Materials and methods

### 2.1 Material

The widely-used cultivar, “Sanying” (*Capsicum annuum* L.) was utilized. It has great disease and pest resistance as well as high production.

### 2.2 Experiment design

**Figure 1:**
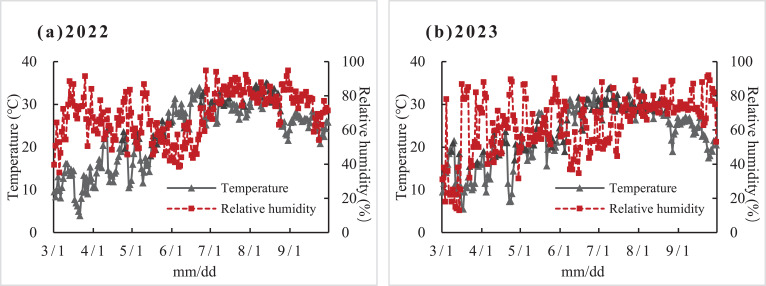
Temperature and relative humidity of environment during the experiments in 2022 **(A)** and 2023 **(B)**.

The Experiments 1, 2 and 3 were conducted at the Agrometeorological Experimental Station of Shangqiu Meteorological Service (34.2°N, 115.6°E, 44 m of elevation) from March to September in 2022 and 2023, respectively. The air temperature and relative humidity during the experiments were showed in **Figure 1**.

The plants were sown in polypropylene plastic pots (21.0 cm × 21.4 cm × 19.1 cm) filled with a vermiculite: substrate: perlite mixture of 1:1:1 (v: v: v). There are no holes at the bottom of plastic basins to prevent water loss due to gravity. The water capacity of each pot was kept within a set range by weighing with electronic scale (Lichen YP300001D, accuracy of 0.1 g) at 7:00 and 18:00 every day. There were four water capacities (75–85%, 65–75%, 55–65% or 45–55%) and four treatment days (2, 4, 6 or 8 days) were therefore designed for Exp. 1 & 2 (**Table 1**). The data from Exp. 1 were used to construct models, while the data from Exp. 2 were used to validate and screen out the optimal simulations. Plants were grown at water capacities of 70–80%, 60–70% or 50–60% over 3, 5 or 7 days for model validation (Exp.3, **Table 2**). The water capacities and treatment durations used were the median values from the treatments in Exp. 1. The water capacity of 75–85% was set as the control (CK) in all experiments. Thirty plants with healthy and similar growth were selected for each experiment when four true leaves appeared. The water capacities of all the plants were kept at 75–85% before and after the treatments during the experiments. Awnings were used to ensure that the plants would not be wet by rain.

**Table 1:** Treatments used in Experiments 1 & 2.

Water capacity (%)	Days			
	2	4	6	8
75–85%	CK			
65–75%	W1D1	W1D2	W1D3	W1D4
55–65%	W2D1	W2D2	W2D3	W2D4
45–55%	W3D1	W3D2	W3D3	W3D4

**Table 2:** Treatments used in Experiment 3.

Water capacity (%)	Treatment days (d)		
	3	5	7
70%–80%	V1	V2	V3
60%–70%	V4	V5	V6
50%–60%	V7	V8	V9

### 2.3 Methods

#### 2.3.1 Phenology

We recorded the start and end dates of each pepper growth period, including planting, flowering (the first flower with more than 30% of plants blooming), fruit set (the first fruit with more than 30% of plants setting), and harvest stage (all of the pepper fruits turning red) were observed and recorded.

#### 2.3.2 Meteorological data

Air temperature, relative humidity and photosynthetic active radiation were automatically recorded with a FT-QC7-RP sensor every 10 minutes. Averages values per hour or a day were used in the models.

#### 2.3.3 Physiological development time

Physiological development time (PDT) is the cumulative time of growth under the optimum temperature and light (Xu et al., 2020a; Zhang et al., 2022). For a specific cultivar, the cumulative PDT at each growth period is theoretically constant (Cheng et al., 2019; Giolo et al., 2021; Zhang et al., 2022). Cumulative PDT is computed by relative thermal effectiveness (RTE) and relative photoperiod effectiveness (RPE).


(1) $$\text{PDT }=\sum_{\text{i}=1}^{\text{n}}{\text{RTE}}_{\text{i}}\cdot {\text{RPE}}_{\text{i}}$$

where i and n represent the i^th^ day and the total number of days of the plant growth period, respectively.

RTE represents relative growth of a plant at actual temperature for one day relative to those at the optimal temperature.


(2) $$\text{RTE}=\left\{ \begin{array}{l} \;0\text{ T }\leq {\text{ T}}_{\text{b}}\\ (\text{T }-{\text{T}}_{\text{b}})/\left({\text{T}}_{\text{ob}}\;-{\text{ T}}_{\text{b}}\right)\;\;\;{\text{T}}_{\text{b}}<\text{ T }<{\text{ T}}_{\text{ob}}\\ \;1\;{\text{T}}_{\text{ob}}\;\leq \text{ T }\leq {\text{ T}}_{\text{ou}}\\ \left({\text{T}}_{\text{m}}\;-\text{ T}\right)/\left({\text{T}}_{\text{m}}\;-{\text{ T}}_{\text{ou}}\right)\;\;\;\;\;{\text{T}}_{\text{ou}}\;<\text{ T }<{\text{ T}}_{\text{m}}\\ \;0\text{ T}\geq {\text{T}}_{\text{m}} \end{array}\right.$$

where T represents the actual temperature of environment, T_m_ and T_b_ represent upper and lower limit temperatures, and T_ou_ and T_ob_ represent upper and lower limit of optima. Three critical temperatures at various stages of peppers are presented in **Table 3** (Yue et al., 2018).

**Table 3:** The three critical temperatures for the growth of peppers.

Growth period	Lower temperature (°C)	Optimum temperature (°C)	Upper temperature (°C)
Seedling	10	25	35
Flowering	15	20	35
Fruit setting	15	25	35

RPE represents the growth of plants under an actual photoperiod for one day relative to that under the optimal photoperiod.


(3) $$\text{RPE}=\{\begin{array}{cc} 0 & \text{DL}\geq {\text{DL}}_{\text{c}}\\ \left(\text{DL}-{\text{DL}}_{\text{c}}\right)/\left({\text{DL}}_{\text{o}}-{\text{DL}}_{\text{c}}\right) & {\text{DL}}_{\text{o}}<\text{DL}\leq {\text{DL}}_{\text{c}}\\ 1 & \text{DL}\leq {\text{DL}}_{\text{o}} \end{array}$$

where DL_c_ denotes the critical day-length of peppers (16 hours), and DL_o_ denotes the optimal day-length (10 hours). DL represents the actual day-length:


(4) $$\text{DL}=12\times [1+\frac{2}{\pi }\cdot \text{asin }\left(\text{a}/\text{b}\right)$$

(5) a=sinλ×sinδ

(6) b=cosλ×cosδ

(7) sinδ=−sin (π×23.45/180×cos (2π×(DOY+10)/365))

(8) $$cos\delta =\;\sqrt{1-sin\delta \times sin\delta }$$

where λ denotes the latitude of the study region (34°45’ N), δ denotes the obliquity of the ecliptic, and DOY is day of the year.

#### 2.3.4 Product of thermal effectiveness and PAR

The product of thermal effectiveness and photosynthetically active radiation (PAR), which is defined as TEP, can be used to model growth (Wu et al., 2021). Cumulative TEP is obtained from the accumulation of daily relative TEP (DTEP):


(9) $$\text{TEP }=\sum_{i=1}^{n}DTEP_{i}=\sum_{i=1}^{n}(RTE_{i}\cdot PAR_{i})$$

where DTEP_i_ (MJ·m^-2^), RTE_i_ (MJ·m^-2^) and PAR_i_ (MJ·m^−2^·d^−1^) represent TEP, daily mean thermal effectiveness and PAR of the i^th^ day, and n denotes the total number of days of the period.

#### 2.3.5 Growing degree day

GDD is used to express the relationship between effective accumulated temperature and development (Wu et al., 2021). Only temperature is required in the calculation. GDD is the summation of effective temperature, i.e., the cumulative difference between daily mean temperature and lower limit temperature:


(10) $${\text{T}}_{\text{avg}}=\{\begin{array}{c} \;\;\;\;\;\frac{{\text{T}}_{\text{x}}+{\text{T}}_{\text{n}}}{2}\;\;\;\;\;\;\;\;\;\;\;\;{\text{T}}_{\text{base}}\leq {\text{T}}_{\text{avg}}\leq {\text{T}}_{\text{upper}}\\ \;{\text{T}}_{\text{base}}\;\;\;\;\;\;\;\;\;\;\;\;\;\;\;\;\;\;\;{\text{T}}_{\text{avg}}\leq {\text{T}}_{\text{base}}\\ \;\;\;{\text{T}}_{\text{upper}}\;\;\;\;\;\;\;\;\;\;\;\;\;\;\;\;\;{\text{T}}_{\text{avg}}\geq {\text{T}}_{\text{upper}} \end{array}$$

(11) $$\text{GDD }=\;\sum_{i=1}^{n}(T_{avg}-T_{base})$$

where T_avg_ represents daily mean temperature (°C), T_n_ represents daily minimum temperature (°C), T_x_ represents daily maximum temperature (°C), T_upper_ and T_base_ represent the upper and lower limit temperature (°C), respectively, and n represents the total number of days of the period.

#### 2.3.6 Interpolation methods

Based on MATLAB R2018a, the five interpolation methods, including linear, nearest, cubic, makima and spline, were used to construct growth models under various field water deficits of 45%–85% over 2–8 days.

#### 2.3.7 Model construction and validation

Based on the results of Exp. 1, the PDT, TEP and GDD models were used to simulate pepper growth. The meteorological data and phenology from Exp. 2 were used to validate and screen out the optimal method according to the root mean squared error (RMSE), relative error (RE), and coefficient of determination (R^2^) (Tang et al., 2007; Shi et al., 2022). The optimal simulation method was selected to construct models of growth based on values of RMSE, RE and R^2^.


(12) $$\text{RMSE }=\sqrt{\frac{\sum_{\text{i}=1}^{\text{n}}{\left({\text{OBS}}_{\text{i}}-{\text{SIM}}_{\text{i}}\right)}^{2}}{\text{n}}}$$

(13) $$\text{RE }\left(\%\right)=\frac{\text{RMSE}}{\sum_{\text{i}=1}^{\text{n}}{\text{OBS}}_{\text{i}}}\cdot \text{n}\times 100$$

where OBS_i_ and SIM_i_ represent observed and simulated values, and n is the number of samples.


(14) $${\text{R}}^{2}=1-\left(\text{residual SS}/\text{corrected SS}\right)$$

where residual SS denotes residual sum of squares and corrected SS denotes corrected sum of squares.

## 3 Results

### 3.1 Effect of water deficits on growth

Under the optimal condition (CK), it took 96, 116 and 171 days from planting to flowering, fruit set and harvest, respectively (**Table 4**). The plants under W1D1, W1D2, W1D3, W2D1 and W2D2 were advanced compared with the controls. Days to flowering, fruit set, and harvest under W1D3 were 3, 4 and 5 days earlier. However, the plants under water capacity of 55–65% for 6 or 8 days, or water capacity of 45–55% for 2, 4, 6, 8 days were all delayed. Days to flowering, fruit set, and harvest under W3D4 were 12, 14 and 18 days slower than the controls.

**Table 4:** Effect of water deficits on the phenology of pepper in Exp. 1 (N=30).

Treatment	Days to flowering (d)	Days to fruit set (d)	Days to harvest (d)
CK	96	116	171
W1D1	94	114	169
W1D2	94	113	168
W1D3	93	112	166
W1D4	97	118	172
W2D1	94	113	168
W2D2	93	112	168
W2D3	98	119	173
W2D4	99	120	174
W3D1	101	123	175
W3D2	103	125	181
W3D3	105	127	185
W3D4	108	130	189

### 3.2 Simulation of growth with PDT, TEP and GDD

**Figure 2:**
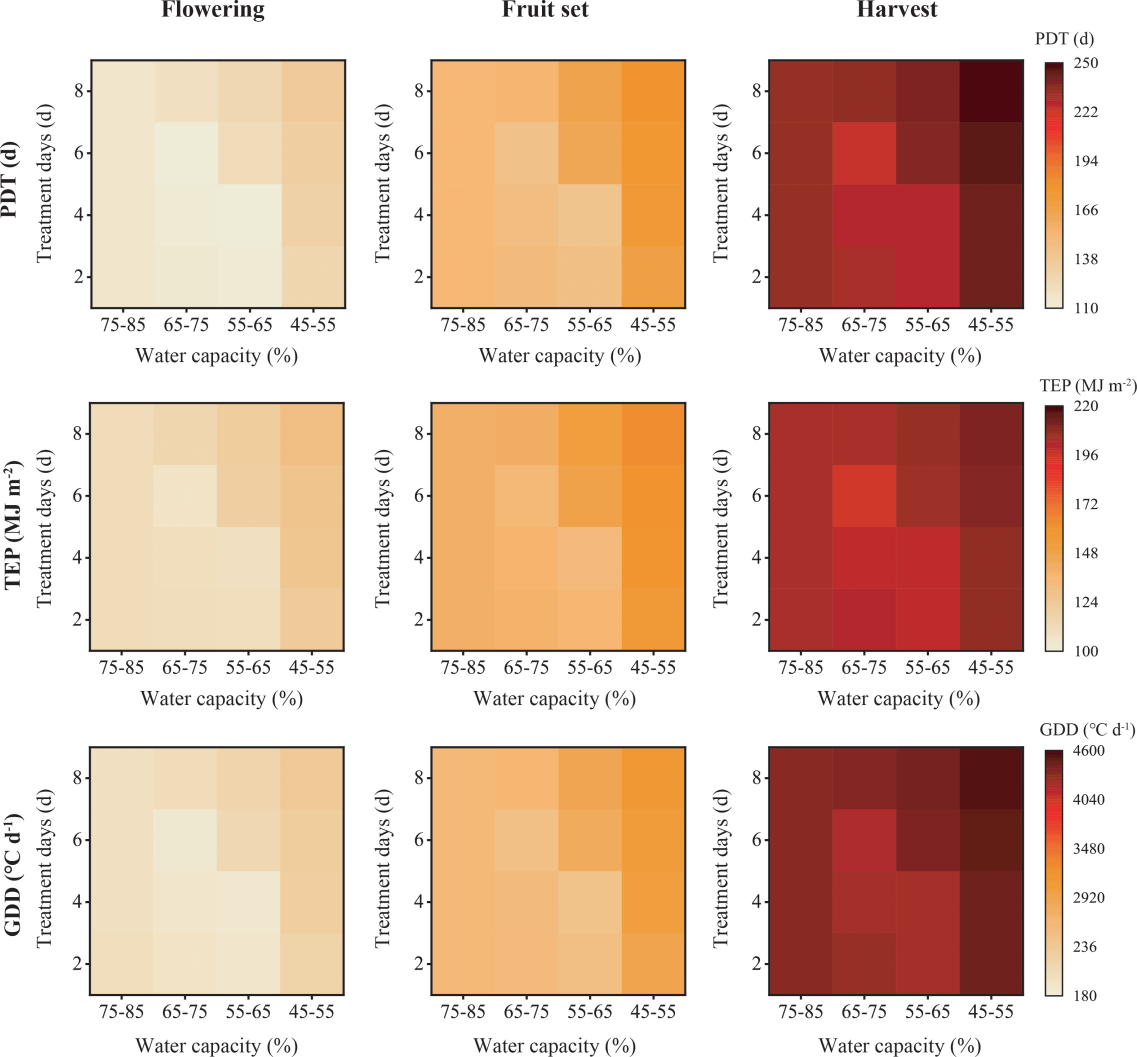
Cumulative physiological development time (PDT), product of thermal effectiveness and PAR (photosynthetically active radiation) (TEP) and growing degree days (GDD) required for pepper plants to flowering, fruit set and harvest under different water deficits (N=30).

Phenology of the plants is shown in **Figure 2**, with the darker the color, the slower growth. Cumulative PDT/TEP/GDD under water capacity of 75–85% for 2, 4, 6 or 8 days were similar. Growth was accelerated for W1D1, W1D2, W1D3, W2D1 and W2D2 (lighter). Plants given a water capacity of 65–75% for 6 days had the quickest growth. However, plants given a water capacity of 55–65% for 6 or 8 days, or a water capacity of 45–55% for 2, 4, 6 or 8 days were slower. Plants given a water capacity of 45–55% for 8 days required higher values of cumulative PDT, TEP, and GDD than the other plants.

### 3.3 Simulation and validation of growth models using PDT, TEP or GDD

**Figure 3:**
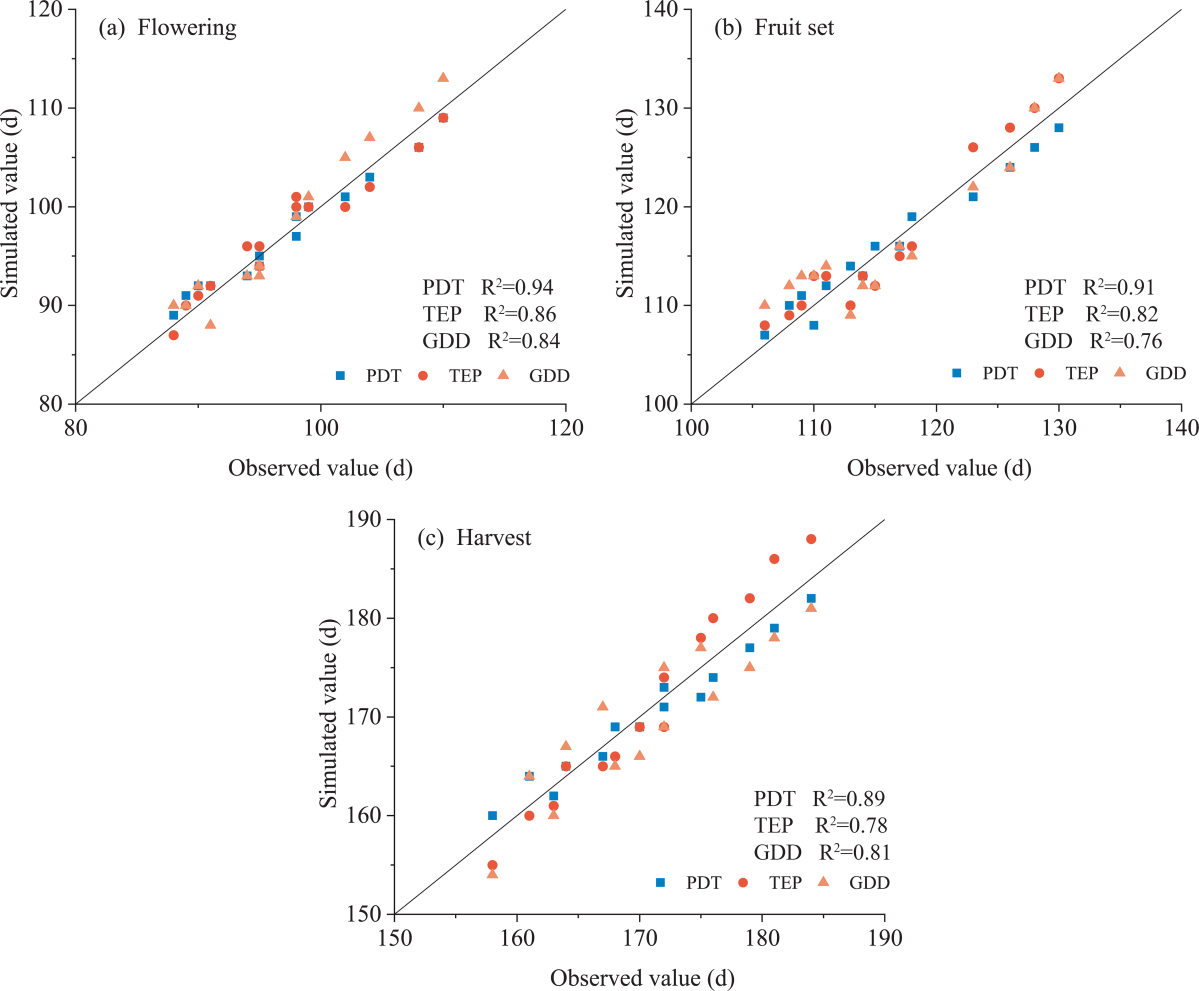
Comparison of observed and simulated days to flowering **(A)**, fruit set **(B)** and harvest **(C)** in pepper with physiological development time (PDT), product of thermal effectiveness and PAR (photosynthetically active radiation) (TEP) and growing degree days (GDD) models. Dashed line represents the 1:1 line.

All of three methods accurately simulated growth with the scatter points of the data nearly distributed along the 1:1 line (**Figure 3**). Overall, the scatter for PDT was closer to the 1:1 line than that for other two methods. Simulation with PDT showed the highest R^2^ of 0.94, 0.91, and 0.89 for flowering, fruit set, and harvest compared with TEP and GDD. The TEP model had a higher R^2^ than GDD in simulating flowering (0.86) and fruit set (0.82), with a smaller R^2^ in simulating harvest (0.78).

Simulation errors increased as growth increased (**Table 5**). For example, the errors from planting to flowering with PDT, TEP or GDD were 0–2, 1–3 or 1–3 days. The errors from planting to harvest using PDT, TEP or GDD increased to 1–3, 1–5 or 2–4 days. Simulations using PDT were overall better with smaller errors. Simulations using PDT with water capacity of 75–85% were more accurate than those for the other treatments with the errors only 1 or less day for three growth periods. The GDD models had a larger error in simulating growth (mostly 3–4 days).

**Table 5:** Simulated errors (observed - simulated values) for the number of days from planting to flowering, fruit set, and harvest of peppers using models based on physiological development time (PDT), product of thermal effectiveness and PAR (photosynthetically active radiation) (TEP) and growing degree days (GDD).

Period	Water capacity	PDT				TEP				GDD			
		Days				Days				Days			
		2	4	6	8	2	4	6	8	2	4	6	8
Planting to flowering	75–85% 65–75% 55–65% 45–55%	95 (0) 93 (1) 92 (-2) 101 (1)	94 (1) 92 (-1) 89 (-1) 103 (1)	95 (0) 91 (-2) 99 (-1) 106 (2)	94 (1) 97 (1) 100 (-1) 109 (1)	94 (-1) 96 (2) 91 (1) 100 (-2)	96 (1) 92 (1) 87 (-1) 102 (-2)	94 (-1) 90 (1) 101 (3) 106 (-2)	96 (1) 100 (2) 100 (1) 109 (-1)	93 (-2) 93 (-1) 92 (2) 105 (3)	94 (-1) 88 (-3) 90 (2) 107 (3)	93 (-2) 90 (1) 99 (1) 110 (2)	94 (-1) 99 (1) 101 (2) 113 (3)
Planting to fruit set	75–85% 65–75% 55–65% 45–55%	114 (-1) 112 (-1) 108 (2) 121 (2)	113 (1) 111 (-2) 107 (-1) 124 (2)	114 (-1) 110 (-2) 116 (-1) 126 (2)	113 (1) 116 (1) 119 (-1) 128 (2)	110 (-3) 113 (2) 113 (3) 126 (3)	113 (-1) 110 (1) 108 (2) 128 (2)	110 (-3) 109 (1) 112 (-3) 130 (2)	113 (-1) 115 (-2) 116 (-2) 133 (3)	109 (-4) 114 (3) 113 (3) 122 (-1)	112 (-2) 113 (4) 110 (4) 124 (-2)	109 (-4) 112 (4) 112 (-3) 130 (2)	112 (-2) 116 (-1) 115 (-3) 133 (3)
Planting to harvest	75–85% 65–75% 55–65% 45–55%	169 (1) 166 (1) 162 (1) 174 (2)	169 (-1) 165 (-1) 160 (-2) 177 (2)	169 (1) 164 (-3) 171 (1) 179 (2)	169 (-1) 173 (-1) 172 (3) 182 (2)	169 (-1) 165 (-2) 161 (-2) 180 (4)	166 (-2) 165 (1) 155 (-3) 182 (3)	169 (-1) 160 (-1) 174 (2) 186 (5)	166 (-2) 169 (-3) 178 (3) 188 (4)	166 (-4) 171 (4) 160 (-3) 172 (-4)	165 (-3) 167 (3) 154 (-4) 175 (-4)	166 (-4) 164 (3) 175 (3) 178 (-3)	165 (-3) 169 (-3) 177 (2) 181 (-3)

N=30.

**Figure 4:**
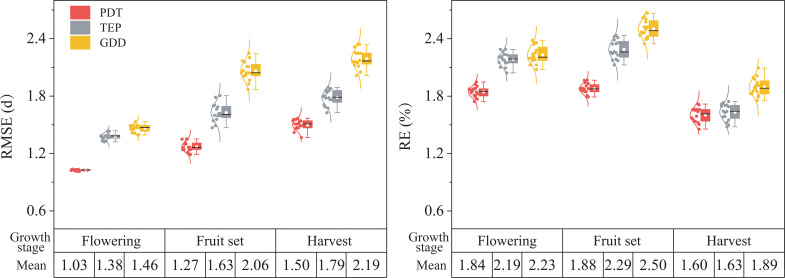
Boxplots of RMSE (d) and RE (%) for the simulation of growth in pepper from planting to flowering, fruit set, and harvest, with physiological development time (PDT), product of thermal effectiveness and PAR (photosynthetically active radiation) (TEP) and growing degree days (GDD) models under different water deficits.

Overall, the RMSE values of simulations with PDT, TEP, and GDD increased with growth, while the RE of the simulations increased and then decreased (**Figure 4**). The simulation of peppers from planting to flowering with PDT had the lowest RMSE (average of 1.03 d) compared with other methods. However, the mean RE of simulation for planting–harvest was 0.24 and 0.28 lower than that for planting–flowering and planting–fruit set. The GDD model had the highest RMSE and RE in simulating growth.

### 3.4 Construction of growth models with five interpolation methods

**Figure 5:**
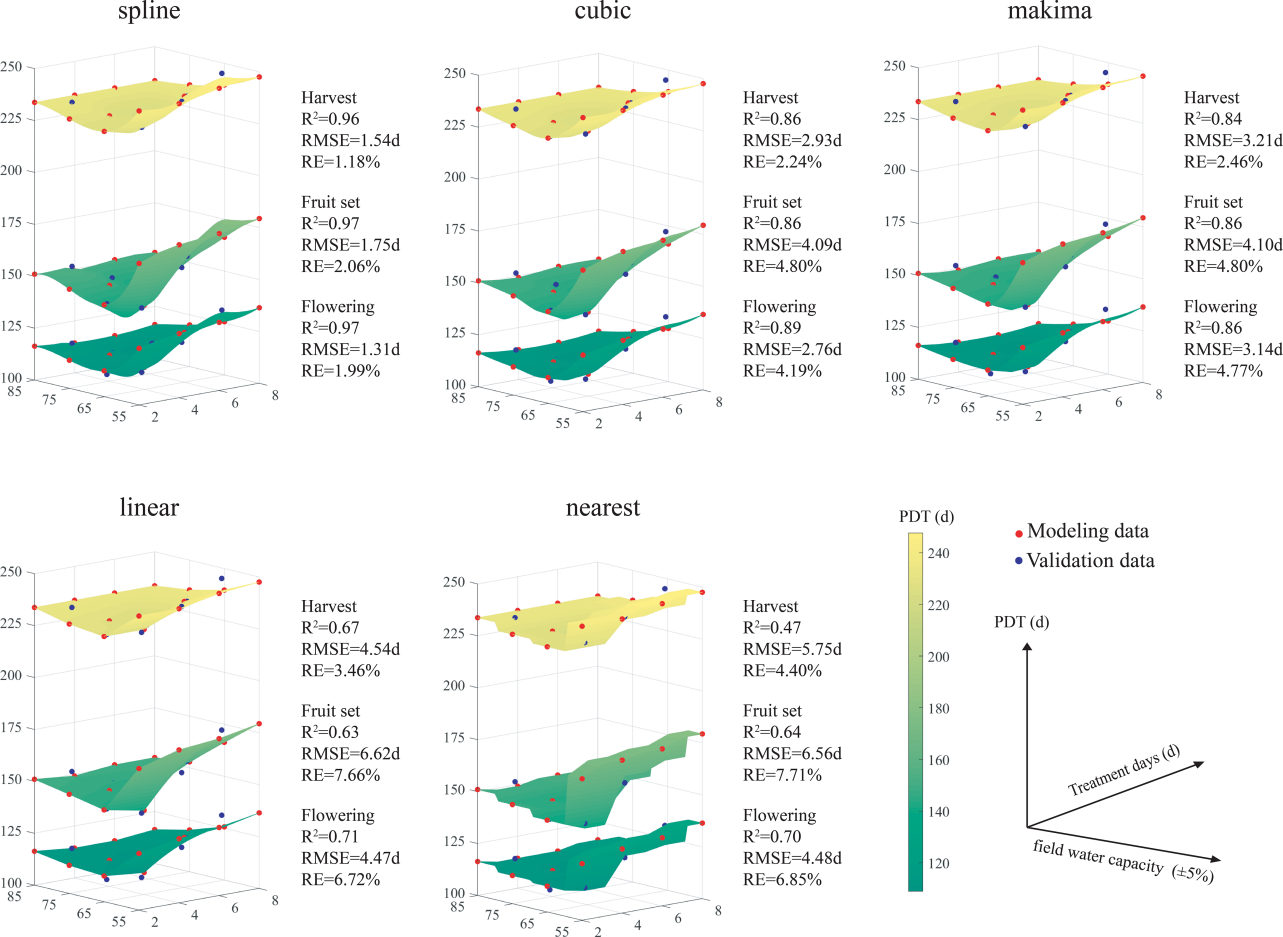
Three-dimension surface map of flowering models in pepper under water deficits with spline, cubic, makima, linear and nearest interpolation algorithms based on accumulated physiological development time (PDT).

The PDT model was more accurate to predict growth and was selected for the simulation study with water capacities of 45–85% over 2–8 days (red scatter points in **Figure 5**). This experiment then compared and validated the distinct models (blue scatter points in **Figure 5**), based on values of RMSE, RE and R^2^.

All the models exhibited similar trends under water deficits. Cumulative PDT required for each growth period showed a decreased trend and then an increased trend as water capacity decreased from 85% to 45%. The required PDT of each growth period remained constant under water capacity of 75–85% over 2–8 days, and increased under water capacity of 45–55% over 2–8 days.

The interpolation surfaces for the spline, cubic and makima models were smoother than that of the linear or nearest models. Overall, the validations were better using spline than using the other models. The spline models had the highest R^2^ (0.96–0.97) and the lowest RMSE (1.31–1.75 d) and RE (1.18–2.06%). The errors with spline were in the order of flowering< fruit set< harvest. The RMSE and RE of each pepper growth period using nearest model were the highest among the five models, except for a slight lower RMSE (0.06 d) than the linear model in planting–fruit set stage. The interpolation images of nearest models for growth were segmentate.

## 4 Discussion

Pepper is a typical short-day plant (Lu et al., 2020; Zhang et al., 2020; Wang et al., 2021). The PDT model characterized the relationship between temperature and development speed by converting the response of crop to temperature into relative thermal effectiveness on the basis of three critical points of temperature, but also considered the relative photoperiod effectiveness of crops (Zhu et al., 2023). Thus, this model was more accurate and robust than those based on TEP and GDD.

Combined stresses of different water capacities and duration days might affect the growth differently. Interpolation models can approximately simulate growth under various water capacity and duration days. Spline models gave the highest values of R^2^ and the lowest RMSE and RE across all of the growth. This response is consistent with the results of Gore et al. (2023). Development with water capacities of 75–85% for 2, 4, 6, and 8 days was similar possibly, because 75–85% is the optimal water level for peppers. Phenology was accelerated as water capacity decreased from 85% to 45%, and then was delayed. Cumulative PDT for each growth period under water capacity of 65–75% for 2–6 days and 55–65% for 2–4 days was lower than that under the other treatments, indicating a mild water deficit may promote crop growth and development (Xu et al., 2020b; Zhang et al., 2023b). However, long-term mild and moderate water deficits (65–75% for 8 days or 55–65% for 6–8 days) and severe water deficits (45–55% for 2–8 days) can significantly decrease growth, protein metabolism and yield (Jiang et al., 2008; Li et al., 2020; Yang et al., 2022).

The spline model was proved to successfully and precisely simulate growth under a water capacity from 45% to 85% for 2–8 days based on accumulated PDT. Nevertheless, some limitations exist in our study.

The size of pots used to plant peppers in this study was based on earlier research (Zhang et al., 2023b; Zhu et al., 2023). However, previous studies have demonstrated that root restrictions can reduce growth (He et al., 2022; Li et al., 2022; Liu et al., 2023). The applicability of the models in our study may not apply to other cultivars and growing system.

We only used water capacities of 45–85% over 2–8 days, which is common in commercial pepper cultivation. Further, studies are required with other watering strategies (Mills et al., 2018; Clifton et al., 2020; Otieno et al., 2022).

## 5 Conclusion

Three independent experiments were conducted to construct and validate models in pepper growth under water deficits. Mild water deficits (65–75% for 2–6 days or 55–65% for 2–4 days) accelerate development while severe water deficits (65–75% for 8 days, 55–65% for 6–8 d or 45–55% for 2–8 days) delay development. Models based on PDT simulated growth from planting to flowering, fruit set, and harvest compared those based on TEP and GDD. Growth can be simulated using spline interpolation under a water deficit based on a water capacity from 45% to 85% over 2 to 8 days. The study provides a scientific basis for assessment of pepper growth and development under various water deficits to develop irrigation strategies for best commercial production.

## Data Availability

The original contributions presented in the study are included in the article/supplementary material. Further inquiries can be directed to the corresponding author.
